# Retraction: Gadd153 and NF-κB Crosstalk Regulates 27-Hydroxycholesterol-Induced Increase in BACE1 and β-Amyloid Production in Human Neuroblastoma SH-SY5Y Cells

**DOI:** 10.1371/journal.pone.0307676

**Published:** 2024-07-18

**Authors:** The PLOS ONE Editors

After this article [1] was published, concerns were raised about results presented in Figures 1, 3, and 4. Specifically:

- The p50 panel in Fig 1A in [1] appears similar to the CHOP panel in Fig 2C in [2].
- In the BACE1 panel in Fig 1C, lanes 1, 2, and 8 appear similar to each other, and lanes 3 and 9 appear similar to each other.
- In the β-Actin panel in Fig 1C, there appears to be a vertical discontinuity in the background between lanes 7 and 8 suggestive of possible image splicing.
- In the Gadd153 panel in Fig 3A:
  - Lane 1 appears similar to lane 7, lane 2 appears similar to lanes 8 and 11, and lane 3 appears similar to lanes 9, 10, and 12.
  - There appears to be a vertical discontinuity in the background between lanes 6 and 7 suggestive of possible image splicing.
- In the β-Actin panel in Fig 3A:
  - Lanes 6–10 appear similar to lanes 1–5 in the β-actin panel in Fig 4A, lanes 11–12 appear similar to lanes 11–12 in the β-actin panel in Fig 4A, and lanes 1–5 appear similar to lanes 6–10 in the β-actin panel in Fig 4A.
  - There appears to be a vertical discontinuity in the background between lanes 5 and 6 suggestive of possible image splicing.

The authors did not respond to queries about the experiments in Figures 1, 3, and 4.

In light of the extent and nature of the concerns listed above that question the reliability of the reported results and conclusions, the *PLOS ONE* Editors retract this article.

All authors either did not respond directly or could not be reached.

Owing to the concerns about similarities with previously published content [2], published in 2011 by Elsevier B.V., which is not offered under a CC BY license, the p50 panel in Figure 1A is excluded from this article’s [1] license. At the time of retraction, the article [1] was republished to note this exclusion in the legend of Fig 1 and the article’s copyright statement.
